# MiR-23c Regulates the Resistance to Gefitinib in EGFR Mutant Non-Small-Cell Lung Cancer Cells

**DOI:** 10.3390/cells15121043

**Published:** 2026-06-06

**Authors:** Brigitta Ignoto, Ilaria Assunta Parisi, Cristin Roma, Rosa Camerlingo, Serena Dotolo, Salvatore Tufano, Monica Rosaria Maiello, Nicola Normanno, Alessandro Morabito, Antonella De Luca, Daniela Frezzetti

**Affiliations:** 1 Cell Biology and Biotherapy Unit, Istituto Nazionale Tumori-IRCCS-Fondazione G. Pascale, 80131 Naples, Italy; brigitta.ignoto@istitutotumori.na.it (B.I.); ilariaassunta.parisi@istitutotumori.na.it (I.A.P.); c.roma@istitutotumori.na.it (C.R.); r.camerlingo@istitutotumori.na.it (R.C.); serena.dotolo@istitutotumori.na.it (S.D.); salvatore.tufano@istitutotumori.na.it (S.T.); m.maiello@istitutotumori.na.it (M.R.M.); d.frezzetti@istitutotumori.na.it (D.F.); 2 Center for Advanced Molecular Diagnostics in Oncology, Fondazione Policlinico Universitario Agostino Gemelli IRCCS, 00168 Rome, Italy; nicola.normanno@policlinicogemelli.it; 3 Thoracic Department, Istituto Nazionale Tumori-RCCS-Fondazione G. Pascale, 80131 Naples, Italy; a.morabito@istitutotumori.na.it

**Keywords:** lung cancer, EGFR, EGFR-TKIs, microRNA, gefitinib resistance

## Abstract

Mechanisms of primary and acquired resistance are responsible for treatment failure with the Epidermal Growth Factor Receptor-Tyrosine Kinase Inhibitors (EGFR-TKIs) in the majority of patients with advanced Non-Small-Cell Lung Cancer (NSCLC) carrying EGFR-activating mutations. MicroRNAs (miRNAs) are important modulators of EGFR signaling in lung cancer. Recent studies suggested the role of miR-23c as a tumor suppressor or oncogenic miRNA in different tumor types. However, the role of miR-23c in NSCLC carrying EGFR mutations and its involvement in resistance to EGFR-TKIs has not been explored yet. We found that miR-23c was strongly downregulated in H1975 and HCC827-Gefitinib-Resistant (GR) NSCLC cell lines with intrinsic and acquired resistance to gefitinib, respectively, as compared to gefitinib-sensitive cell lines. Moreover, we demonstrated that miR-23c mimic inhibited proliferation, migration, invasion, and epithelial–mesenchymal transition of resistant cells and that Interleukin-6 Receptor (IL-6R) is a direct target of miR-23c in H1975 and HCC827-GR cell lines. Importantly, miR-23c mimic re-sensitized NSCLC-resistant cells to gefitinib, whereas the combination of miR-23c mimic with a neutralizing IL-6R antibody potentiated the sensitivity to the drug. Collectively, our data demonstrated that miR-23c acts as a tumor suppressor in NSCLC cell lines carrying EGFR mutations and that the axis miR-23c/IL-6R might represent a potential target for the development of therapeutic approaches to overcome resistance to gefitinib.

## 1. Introduction

Lung cancer is the most frequent tumor type diagnosed in the world (12.7%) and represents the leading cause of cancer-related mortality (18.7%) [1]. Non-Small-Cell Lung Cancer (NSCLC) is the most common type of lung cancer, accounting for ~85% of cases, and includes adenocarcinoma as the major histological subtype [2].

The role of the Epidermal Growth Factor Receptor (EGFR) signaling in lung cancer has been well established [3]. Activating mutations in the tyrosine kinase domain of the EGFR result in ligand-independent activation of EGFR downstream signaling pathways. EGFR-Tyrosine Kinase Inhibitors (TKIs) represent the standard first-line treatment of patients with advanced NSCLC carrying EGFR-activating mutations [4]. However, mechanisms of primary (i.e., intrinsic) and secondary (i.e., acquired) resistance are responsible for treatment failure [5]. In this regard, mechanisms of intrinsic resistance to EGFR-TKIs include EGFR Exon20 insertions, MET amplifications, TP53 mutations, and mutations in cell cycle-related genes. Secondary EGFR mutations (T790M, C797S), activation of alternative bypass signaling pathways (MET, HER2), and histological transformation are well-characterized mechanisms of acquired resistance to these agents [6]. However, other molecular mechanisms might be involved in resistance to EGFR-TKIs and highlighting such mechanisms might help to develop novel therapeutic strategies to overcome resistance.

MicroRNAs (miRNAs) are non-coding, single-stranded RNA molecules of approximately 19–25 nucleotides responsible for post-transcriptional gene regulation. MiRNAs downregulate gene expression by binding to their target mRNAs and promoting their degradation or translation repression, and are involved in several biological processes, such as cell proliferation, differentiation, and apoptosis [7,8]. In addition, miRNAs are frequently deregulated in human cancers and exert their function through the promotion of oncogenic pathways or the repression of tumor-suppressive functions [8].

Recent evidence suggests that altered miRNA expression plays an important role in lung cancer development and progression. Modulation of key miRNAs can affect oncogenic pathways, potentiating the effects of anti-cancer drugs [9,10]. In addition, some studies demonstrated that miRNAs are involved in the regulation of EGFR signaling in NSCLC, modulating cell proliferation, invasion, and survival [11]. In addition, different studies showed that the EGFR mutational status might affect the expression of several miRNAs [10].

In recent years, the role of miR-23c as a tumor suppressor or oncogenic miRNA has been reported in breast, hepatic, and colon cancer. In particular, we, and other authors, demonstrated that miR-23c had a tumor-suppressive role in aggressive breast cancers [12,13]. In addition, it has been observed that miR-23c mimic inhibited cell proliferation and induced apoptosis of hepatocarcinoma cell lines [14]. In endometrial cancer, miR-23c mimic decreased cancer cell invasion and migration [15]. On the other hand, secreted miR-23c levels were increased in prostate cancer cells [16], whereas in colorectal cancer patients, higher miR-23c expression was associated with reduced recurrence-free survival, suggesting an oncogenic role of such miRNA [17].

Starting from this evidence, we investigated the role of miR-23c in NSCLC cell lines with EGFR-activating mutations and analyzed the mechanisms through which miR-23c modulated the resistance to the EGFR-TKI gefitinib in NSCLC cell lines with intrinsic and acquired resistance to the drug.

## 2. Materials and Methods

### 2.1. Cell Cultures

Human NSCLC cell lines (A549—CVCL_0023, HCC4006—CVCL_1269, HCC827—CVCL_2063, and H1975—CVCL_1511) were purchased from the American Type Culture Collection (ATCC, Manassas, VA, USA) and cultured in RPMI 1640 medium with GlutaMAX supplemented with 10% fetal bovine serum (FBS) (ThermoFisher Scientific, Milan, Italy). HCC827-Gefitinib-Resistant (GR) cells were generated from the HCC827 cell line as previously described [18] and routinely maintained in medium containing 10 nM gefitinib (Cayman Chemical, Ann Arbor, MI, USA).

Normal cells from bronchial epithelium, BEAS-2B (CVCL_0168 from ATCC), were kindly provided by the Department of Pharmacy of the University of Naples Federico II [19] and cultured in DMEM medium with GlutaMAX supplemented with 10% FBS.

All cell lines were maintained in a 5% CO_2_-humidified incubator at 37 °C.

### 2.2. Real-Time PCR

Total RNA was extracted using the TRIzol reagent (Thermo Fisher Scientific). To measure miR-23c expression levels, reverse transcription was performed with the Taqman MicroRNA Reverse Transcription Kit (Thermo Fisher Scientific), according to the manufacturer’s instructions. The TaqMan Universal PCR Master Mix II, no UNG (Thermo Fisher Scientific) and miR-23c probes (Thermo Fisher Scientific) were used for real-time PCR. Relative quantification was performed using the U6 as reference miRNA expression control.

To measure *IL-6R* gene expression, cDNA was synthesized using the SuperScript II Reverse Transcriptase (Thermo Fisher Scientific) and random hexamers as primers, according to the manufacturer’s protocol. Real-time PCR was performed using the QuantStudio 7 Pro (Thermo Fisher Scientific) with the SYBR Green qPCR Master Mix (Thermo Fisher Scientific) and the following primers: 5′ CCCACTCAAAAAGGACACTTCTG 3′ (vimentin forward), 5′ CGTGATGCTGAGAAGTTTCGTT 3′ (vimentin reverse), 5′ TCAACTTGCCAGAAAACTCCAG 3′ (N-cadherin forward), 5′ CCGCAGTGAAAGGTTTTTATCTCT 3′ (N-cadherin reverse), 5′ GCAAATTCCTGCCATTCTGG 3′ (E-cadherin forward), 5′ CGAAGAAACAGCAAGAGCAGC 3′ (E-cadherin reverse), 5′ TGGCCTTCGGAACGCTCCTCT 3′ (IL-6R forward), and 5′ GCCCGCAGCTTCCACGTCTT 3′ (IL-6R reverse). The primers for human GAPDH were purchased from Qiagen (Milan, Italy). Relative quantification was performed using the 2^−∆∆Ct^ method.

### 2.3. Cell Transfection with microRNA Mimics or Inhibitors

MiR-23c mimics (accession no. MIMAT0018000) and miR-23c inhibitors (accession no. MIMAT0018000) were obtained from Thermo Fisher Scientific. We employed commercially available random sequences of miRNA mimics or inhibitors as a non-targeting negative control (NTC) or inhibitor negative control (INC), respectively (Thermo Fisher Scientific).

NSCLC cell lines (1.5 × 10^5^ cells/well) were seeded at 50% confluence into 6-well plates.

Transfection of miR-23c mimics or miR-23c inhibitors and their relative controls was performed using the Lipofectamine RNAiMax Transfection Reagent (Thermo Fisher Scientific), according to the manufacturer’s protocol. After 72 h, transfected cells were harvested for the experiments.

### 2.4. Cell Proliferation and Cell Cycle Analysis

For cell proliferation experiments, transfected cells were seeded into 96-well plates (3 × 10^3^ cells/well) in serum-containing medium. Cells were untreated or treated with gefitinib at different doses with or without 100 ng/mL of anti-IL-6R alpha antibody (R&D Systems, Minneapolis, MN, USA). Cell proliferation was measured using the tetrazolium-based (MTT) colorimetric assay as previously described [12].

For cell cycle analysis, transfected cells were harvested in PBS and fixed in ethanol at 96%. Then, 1 × 10^6^ cells were incubated with 5 µg/mL propidium iodide (Sigma, Chemical Co., St. Louis, MO, USA) plus 25 µL RNAse (1 mg/mL) overnight at 4 °C in the dark. Stained cells were analyzed by the BD FACSAria III Flow Cytometer (Becton, Dickinson and Company, Franklin Lakes, NJ, USA) and the percentage of cells at G0-G1, S and G2-M phases was determined using the BD FACS Diva 8.0 software (Becton, Dickinson and Company).

### 2.5. Migration and Invasion Assays

Cell migration was evaluated using the Colorimetric Cell Migration Assay (Chemicon/Millipore, Milan, Italy). After transfection, 8 × 10^4^ cells were seeded in the upper chambers of a transwell in 0.1% BSA-containing medium. Cells were allowed to migrate for 16 h through the inserts towards the lower chamber containing medium with 10% FBS. Migrating cells were stained with crystal violet, and the absorbance was read at 540 nm.

Cell invasion was measured with the Cell Invasion Assay Kit (Millipore), according to the manufacturer’s instructions. Briefly, transfected cells were seeded in upper chambers (9 × 10^4^ cells) and allowed to invade for 48 h through a membrane coated with matrigel. Medium with 10% FBS was used as a chemoattractant in the lower chamber.

### 2.6. Western Blot Analysis

Western blotting was performed on whole protein extracts, according to standard protocols. We used the following antibodies: anti-vimentin (Abcam, Cambridge, UK), anti-N-cadherin (Abcam), anti-E-cadherin (Santa Cruz Biotechnology, Dallas, TX, USA), anti-IL-6R (Abcam), anti-phospho STAT3 (Tyr705, clone 3E2) (Cell Signaling Technology, Danvers, MA, USA), and anti-STAT3 (Cell Signaling Technology), and anti-α-tubulin clone DM1A (Sigma-Aldrich, Milan, Italy). Densitometric analysis was performed using the ImageJ software (version 1.53e).

### 2.7. Luciferase Reporter Assay

The luciferase reporter plasmid containing the 3′-UTR of IL-6R mRNA with three putative miR-23c binding sites (LUC-IL-6R 3′UTR wt) and the control plasmid containing the 3′-UTR of IL-6R with mutations in two miR-23c binding sites (LUC-IL-6R 3′UTR mt) were obtained as previously described [12]. For luciferase reporter assays, H1975 and HCC827-GR cells were co-transfected with LUC-IL-6R 3′UTR wt/mt plasmids together with miR-23c mimic or NTC. After 72 h, we measured luciferase activity with the Dual-Glo Luciferase Assay System (Promega, Milan, Italy), according to the manufacturer’s instructions. All transfections were performed in triplicate and normalized to Renilla luciferase activity.

### 2.8. Statistical Analysis

Data analysis was performed with GraphPad Prism (version 10, GraphPad Software, San Diego, CA, USA). Data are presented as mean ± standard deviation (SD). Statistical significance was determined using the unpaired two-tailed Student’s *t*-test, and *p* values ≤ 0.05 were considered statistically significant.

## 3. Results

### 3.1. MiR-23c Expression Is Downregulated in EGFR Mutant NSCLC Cell Lines

**Figure 1:**
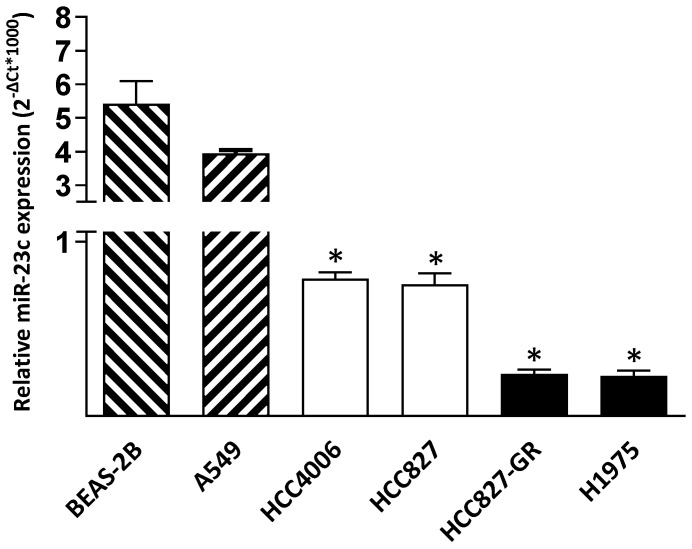
MiR-23c expression in NSCLC cell lines. Real-time PCR analysis of the expression of miR-23c in EGFR mutant (HCC4006, HCC827, H1975 and HCC827-GR) NSCLC cell lines compared to the EGFR wild-type A549 and the BEAS-2B lung normal epithelial cell lines. Data are presented as the mean ± SD from two independent experiments (* *p* < 0.05 for comparison with BEAS-2B cells, two-tailed Student’s *t*-test).

To investigate the role of miR-23c in EGFR-mutant NSCLC cells, we analyzed its levels of expression in EGFR-mutant NSCLC cell lines with different sensitivity to the EGFR-TKI gefitinib, in the EGFR wild-type (wt) A549 cell line, and in the normal lung epithelial cell line BEAS-2B. We found reduced levels of expression of miR-23c in all NSCLC cell lines compared to cells of the normal lung epithelium (Figure 1). We also observed that the HCC4006 cell line carrying the L747-E749del and the A750P mutation and the HCC827 cell line with the E746-A750del, which are highly sensitive to the EGFR-TKI gefitinib [20], had significantly lower levels of expression of miR-23c compared to the EGFR wt A549 cell line. We also analyzed miR-23c expression in EGFRmutant cells with intrinsic and acquired resistance to gefitinib, such as the H1975 cell line, carrying the EGFR-activating L858R mutation together with the T790M resistance mutation, and the HCC827-Gefitinib-Resistant (GR) cell line, previously obtained by prolonged treatment with gefitinib, with an IC50 > 4µM to the drug [18]. Interestingly, the levels of miR-23c expression were strongly downregulated in gefitinib-resistant H1975 and HCC827-GR cells compared to the HCC4006 and HCC827 gefitinib-sensitive cell lines (*p* < 0.005 and *p* < 0.05, respectively, two-tailed Student’s *t*-test), suggesting that miR-23c might have a role in the resistance to EGFR-TKIs in EGFR-mutant NSCLC cells.

### 3.2. MiR-23c Regulates the Proliferation of NSCLC Cell Lines Resistant to Gefitinib

**Figure 2:**
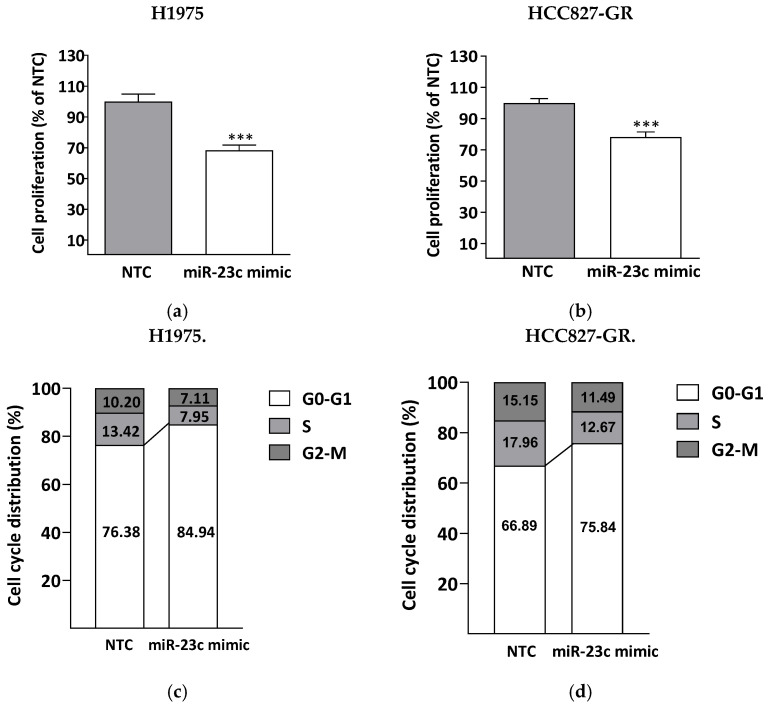
Effect of miR-23c on proliferation and cell cycle distribution in gefitinib-resistant NSCLC cells. Cell proliferation of H1975 (**a**) and HCC827-GR (**b**) cell lines transfected with the miR-23c mimic as compared to their respective control cells transfected with the non-targeting control (NTC) was measured by using an MTT assay 72 h after transfection. Data are presented as the mean ± SD from two independent experiments (n = 8) (*** *p* < 0.0001 for comparison with control cells, two-tailed Student’s *t*-test). Cell cycle distribution of H1975 (**c**) and HCC827-GR (**d**) cell lines transfected with the miR-23c mimic compared to cells transfected with the NTC measured by flow cytometry after staining with propidium iodide. Data are presented as the mean from two independent experiments.

To investigate the functional role of miR-23c in cell lines with intrinsic and acquired resistance to gefitinib, we overexpressed miR-23c in H1975 and HCC827-GR cells. Cell transfection with a miR-23c mimic resulted in a significant increase in miR-23c expression levels in H1975 and HCC827-GR cells compared to cells transfected with the non-targeting control (NTC) (Supplementary Figure S1a,b). The upregulation of miR-23c in H1975 cells produced a decrease in cell proliferation, compared to control cells (Figure 2a). Similarly, in HCC827-GR cells, miR-23c mimic reduced cell proliferation (Figure 2b). Conversely, the downregulation of miR-23c using a miR-23c inhibitor in the gefitinib-sensitive HCC827 cell line produced a significant increase in cell proliferation (Supplementary Figures S1c and S2). Moreover, we observed that H1975 and HCC827-GR cells transfected with the miR-23c mimic showed an increased percentage of cells in G0-G1 phases and a decreased percentage of cells in S and G2-M phases, as compared to cells transfected with the NTC, suggesting that miR-23c inhibited the proliferation of gefitinib-resistant cells through the induction of a G0-G1 cell cycle arrest (Figure 2c,d).

### 3.3. MiR-23c Regulates Migration, Invasion and Epithelial–Mesenchymal Transition Markers in NSCLC Cell Lines Resistant to Gefitinib

**Figure 3:**
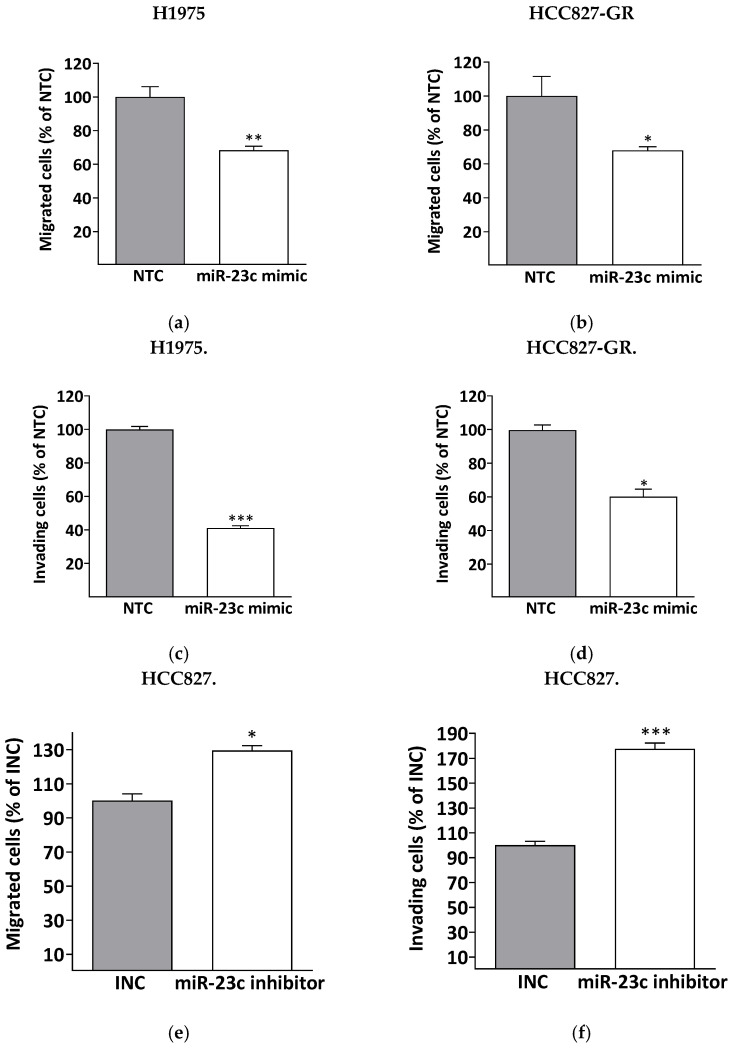
Effect of miR-23c on migration and invasion of NSCLC cells resistant to gefitinib. Migration of H1975 (**a**) and HCC827-GR (**b**) cells transfected with the miR-23c mimic or the NTC. Cells were seeded in transwells 48 h after transfection and were allowed to migrate toward a serum-containing medium for 16 h. Measurement of the invasive ability of H1975 (**c**) and HCC827-GR (**d**) cells transfected with the miR-23c mimic or the NTC was determined by a Boyden chamber-based colorimetric assay 48 h after seeding. (**e**) Migration assay of HCC827 cells transfected with the miR-23c inhibitor or the INC as control. (**f**) Measurement of the invasive ability of the HCC827 cell line transfected with the miR-23c inhibitor or the INC. Statistical significance was determined from three independent experiments (* *p* < 0.05, ** *p* < 0.005, and *** *p* < 0.0001 for comparison with control cells, two-tailed Student’s *t*-test).

We next evaluated whether miR-23c affected the migration and the invasive ability of resistant cell lines using transwell assays. We observed that miR-23c mimic significantly reduced the migration of H1975 and HCC827-GR cells (Figure 3a,b). More importantly, the upregulation of miR-23c produced a significant reduction in cell invasion in gefitinib-resistant cells (Figure 3c,d).

We then analyzed the effects of miR-23 downregulation in HCC827 cells that express higher levels of miR-23c as compared with HCC827-GR and H1975 cells, using a miR-23c inhibitor, and we found that miR-23c downregulation significantly induced cell migration and invasion in HCC827 cells (Figure 3e,f).

Collectively, our data suggested that miR-23c had a suppressive role in NSCLC cells resistant to gefitinib, reducing cell proliferation, migration, and invasion.

**Figure 4:**
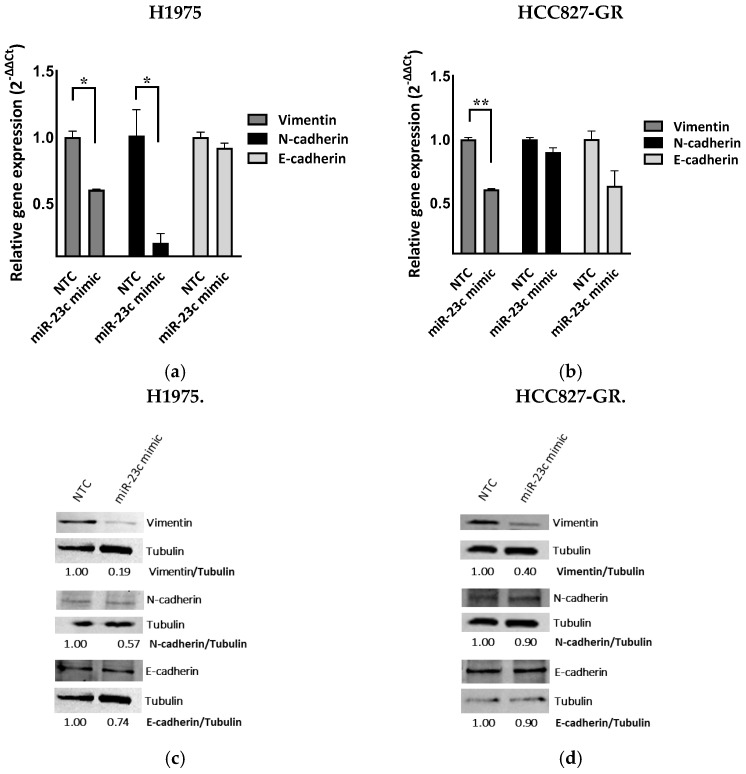
Effect of miR-23c on epithelial–mesenchymal transition in EGFR mutant NSCLC cells. The expression of the mesenchymal markers Vimentin and N-cadherin and of the epithelial marker E-cadherin was evaluated by real-time PCR (**a**,**b**) and Western blot (**c**,**d**) in H1975 (**a**–**c**) and HCC827-GR (**b**–**d**) cells, transfected with the NTC or the miR-23c mimic. Real-time data are presented as the mean ± SD from two experiments (n = 2) (* *p* ≤ 0.05 and ** *p* ≤ 0.005, two-tailed Student’s *t*-test). Blots were normalized with the α-Tubulin antibody.

Since increased cell motility has been associated with epithelial–mesenchymal transition (EMT) [21], we investigated the effects of miR-23c on EMT markers in H1975 and HCC827-GR cells. We observed a significant decrease in transcript and protein expression of the mesenchymal marker vimentin both in H1975 and HCC827-GR cells upon miR-23c transfection compared to control cells. Differently, the expression of the mesenchymal marker N-cadherin was significantly reduced by miR-23c mimic only in the H1975 cell line at both mRNA and protein levels. Finally, only a slight not significant reduction in the epithelial marker E-cadherin was observed in H1975 and HCC827-GR cells transfected with miR-23c mimic (Figure 4a–d). These data suggest a potential involvement of miR-23c in EMT regulation in gefitinib-resistant NSCLC cells.

### 3.4. MiR-23c Targets Interleukin-6 Receptor in Gefitinib-Resistant NSCLC Cells

**Figure 5:**
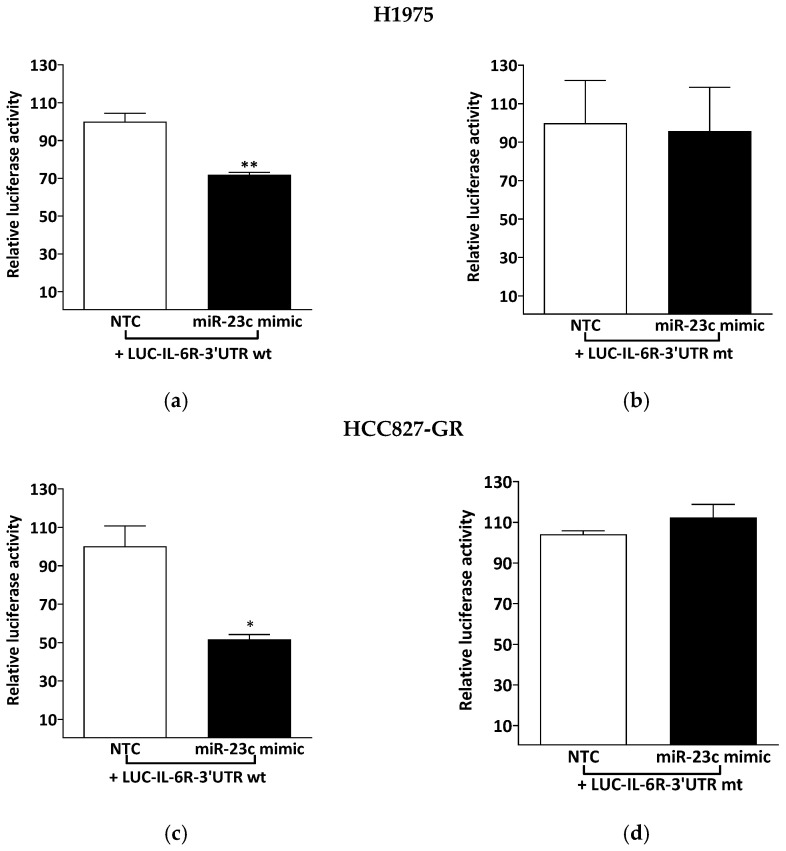
IL-6R is a target of miR-23c in NSCLC cells resistant to gefitinib. Luciferase reporter assays were performed on H1975 (**a**,**b**) and HCC827-GR (**c**,**d**) co-transfected with the miR-23c mimic or the NTC and a luciferase reporter vector containing the IL-6R 3′UTR sequence with wild-type (LUC-IL-6R 3′UTR wt) (**a**–**c**) or mutant (LUC-IL-6R 3′UTR mt) (**b**–**d**) putative miR-23c binding sites. Data are presented as the mean ± SD (n = 3) (* *p* < 0.05 and ** *p* < 0.005 for comparison with cells transfected with the NTC, two-tailed Student’s *t*-test).

To further investigate the molecular mechanisms by which miR-23c exerts its tumor suppressor role in gefitinib-resistant NSCLC cells, we analyzed its potential targets using the MiRWalk 2.0 database. Using an AU score ≥ 0.5, we identified 342 putative candidate target genes selected from TargetScan and miRDB databases included in the MiRWalk 2.0 tool (Supplementary File S1). Among them, we found the Interleukin-6 Receptor (IL-6R) that we previously demonstrated to be regulated by miR-23c in breast cancer cells [12]. To confirm that IL-6R is a target of miR-23c also in gefitinib-resistant NSCLC cells, we co-transfected H1975 and HCC827-GR cells with the miR-23c mimic and a luciferase reporter plasmid containing the 3′-UTR of *IL-6R* with three putative miR-23c binding sites (LUC-IL-6R 3′UTR wt). A mutant luciferase reporter plasmid (LUC-IL-6R 3′UTR mt), containing two mutated miR-23c binding sites, was used as a control. We observed that miR-23c mimic significantly reduced the luciferase activity in resistant cells co-transfected with the LUC-IL-6R 3′UTR wt, but not in cells co-transfected with the LUC-IL-6R 3′UTR mut (Figure 5a–d), confirming that IL-6R is a direct target of miR-23c in gefitinib-resistant NSCLC cells.

**Figure 6:**
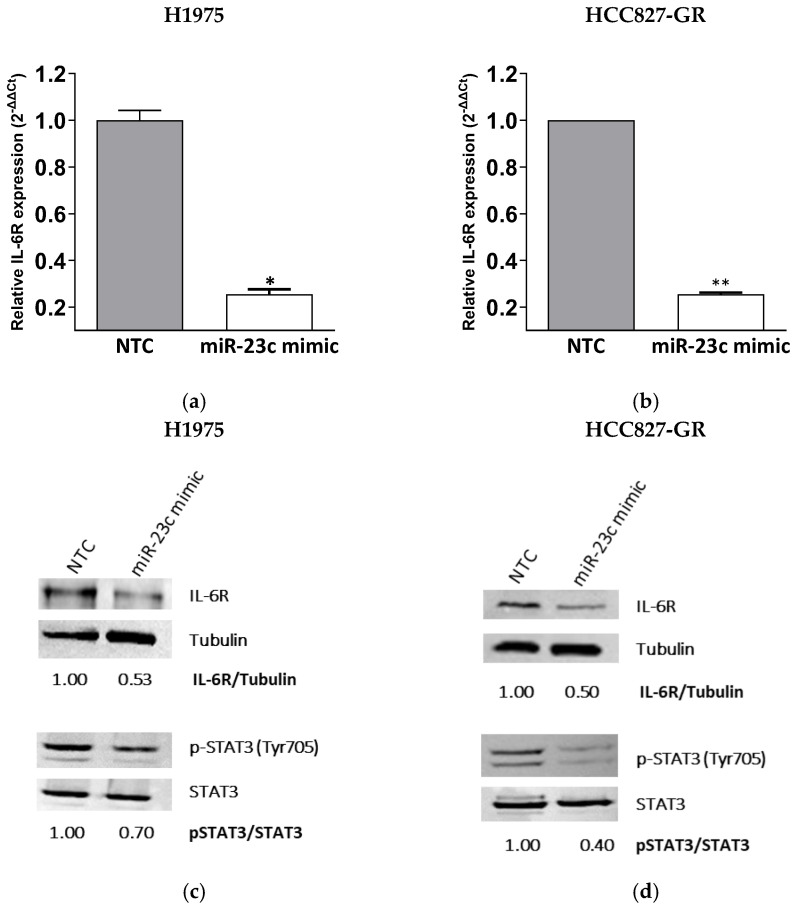
Effects of miR-23c on IL-6R expression and downstream signaling activation in gefitinib-resistant NSCLC cells. The transcript of *IL-6R* in H1975 (**a**) and HCC827-GR (**b**) cells, transfected with the miR-23c mimic or the NTC, was measured by real-time PCR. All quantitative data are presented as the mean ± SD from two independent experiments (* *p* ≤ 0.05 and ** *p* ≤ 0.005, two-tailed Student’s *t*-test). Western blot analysis showing IL-6R and phospho-STAT3 (pSTAT3) protein levels in H1975 (**c**) and HCC827-GR (**d**) cells transfected with the miR-23c mimic or the NTC. Blots were normalized with the α-Tubulin (**upper** panels) and total STAT3 (STAT3) antibodies (**lower** panels), and the relative densitometric values’ ratios for IL-6R/Tubulin and pSTAT3/STAT3 are shown. The original immunoblot images are reported in Supplementary Figure S5.

To evaluate whether miR-23c was able to regulate the expression of the *IL-6R* gene, we analyzed its levels of expression in H1975 and HCC827-GR cells by real-time PCR, and we observed that the transcript of *IL-6R* was reduced in H1975 and HCC827-GR cells transfected with the miR-23c mimic (Figure 6a,b).

In addition, the upregulation of miR-23c significantly decreased the expression of IL-6R protein in H1975 and HCC827-GR cells. Accordingly, the phosphorylation of the downstream effector of IL-6R, STAT3, was significantly reduced in H1975 and HCC827-GR cells transfected with the miR-23c mimic compared to cells transfected with NTC (Figure 6c,d).

### 3.5. MiR-23c Mimic Restores Gefitinib Sensitivity in Resistant NSCLC Cell Lines

**Figure 7:**
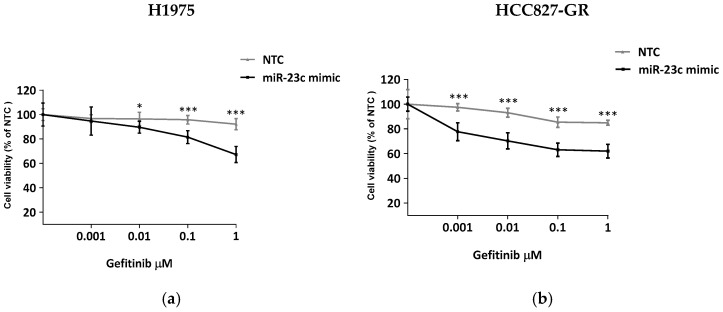
MiR-23c restores the sensitivity to gefitinib in H1975 and HCC827-GR cells. Effect of miR-23c mimic on gefitinib sensitivity in H1975 (**a**) and HCC827-GR (**b**) cells transfected with the miR-23c mimic or the NTC as control. Cell growth was measured by MTT assay after 72 h of treatment with gefitinib at the indicated doses. Data are presented as the mean ± SD from two independent experiments (* *p* < 0.05 and *** *p* < 0.0001 for comparison with control cells, two-tailed Student’s *t*-test).

To assess whether the upregulation of miR-23c might restore the sensitivity to gefitinib, we evaluated cell viability in resistant cells transfected with the miR-23c mimic upon treatment with different concentrations of gefitinib. The miR-23c mimic significantly reduced cell proliferation by 28% (*p* < 0.0001) in H1975 cells as compared to control cells at 1 µM gefitinib (Figure 7a). Similar results were obtained in miR-23c HCC827-GR cells transfected with miR-23c at 1 µM gefitinib (27% reduction as compared to control cells; *p* < 0.0001) (Figure 7b). Conversely, the downregulation of miR-23c decreased the sensitivity to gefitinib of HCC827 transfected with the miR-23c inhibitor (IC50 = ≃ 0.055 µM) compared to control cells transfected with the INC (IC50 = ≃ 0.007 µM) (Supplementary Figure S3). These results suggested that miR-23c might restore the sensitivity to gefitinib in NSCLC cells with acquired or intrinsic resistance to the drug.

**Figure 8:**
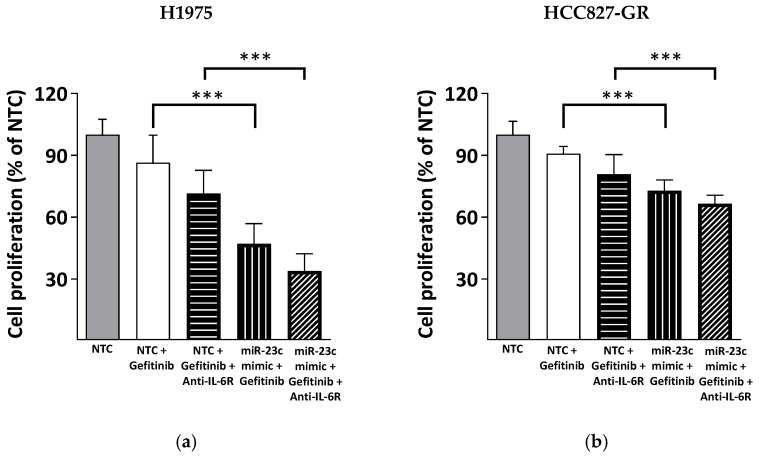
Effect of miR-23c mimic and IL-6R inhibition on gefitinib sensitivity in resistant NSCLC cells. H1975 (**a**) and HCC827-GR (**b**) cells were transfected with the miR-23c mimic or the NTC and treated with a neutralizing anti-IL-6R antibody in the presence of gefitinib 100 nM. Untreated cells transfected with the NTC were used as control. Cell growth was measured by MTT assay after 72 h of treatment. Data are presented as the mean ± SD from two independent experiments (*** *p* < 0.0001 for the indicated comparisons, two-tailed Student’s *t*-test).

Finally, we analyzed whether the blockade of IL-6R further increased the sensitivity to gefitinib in resistant NSCLC cells overexpressing miR-23c. To this aim, we transfected H1975 and HCC827-GR with miR-23c mimic and treated cells with gefitinib and a neutralizing anti-IL-6R antibody. We observed that treatment with gefitinib and the anti-IL-6R antibody produced a significant reduction in cell proliferation of H1975 and HCC827-GR cells transfected with miR-23c mimic, suggesting that the IL-6R blockade in combination with miR-23c mimic potentiated the sensitivity to the drug in resistant NSCLC-cells (Figure 8a,b).

## 4. Discussion

Therapeutic strategies targeting miRNAs that regulate post-transcriptional gene networks involved in tumor progression have been explored as novel approaches to inhibit tumor growth and re-sensitize tumors to anti-cancer agents [10,11]. In this regard, our study provided evidence that miR-23c might be considered a novel therapeutic target in EGFR-mutant NSCLC cells.

MiR-23c has been demonstrated to have a tumor suppressor or oncogenic role in different tumor types [12,13,14,15,16,17]. In lung cancer, data from OncomiR and UALCAN databases revealed that miR-23c is upregulated in lung cancer patients, whereas the dbDEMC database reported that miR-23c can be either upregulated or downregulated in tumors compared to normal tissue in different datasets, suggesting a high heterogeneity of miR-23c functions in lung cancer. Depending on the tumor type, the cellular state, and the genomic background, miRNAs might have different or even opposite functional roles, with their expression being either upregulated or downregulated compared with normal tissue [22,23]. For example, miR-21 has been shown to have different functions in distinct histological subtypes of lung cancer [23]. In addition, it has been observed that miRNAs are differently expressed according to the EGFR mutational status, suggesting that EGFR-activating mutations might regulate miRNA levels. Actually, different studies reported a variety of miRNAs that were differently expressed between the EGFR-mutant and EGFR wt lung adenocarcinoma [24,25,26,27]. However, the role of miR-23c in NSCLC carrying EGFR mutations has not been clarified yet. Here, we showed for the first time that miR-23c was significantly downregulated in EGFR-mutant NSCLC cell lines compared to the EGFR wt A549 cell line. Our results were consistent with data from studies that showed a tumor-suppressive role of miR-23c [12,13,14,15]. More importantly, we demonstrated that miR-23c expression levels were further downregulated in NSCLC cells with intrinsic (H1975) and acquired (HCC827-GR) resistance to the EGFR-TKI gefitinib, acting as a tumor suppressor by negatively regulating cell proliferation, migration, and invasion. However, further studies are required to investigate whether miR-23c might play a role in other mechanisms of resistance to targeted agents in NSCLC, such as MET amplification or small-cell transformation.

Importantly, we provided the first evidence that a miR-23c mimic can restore sensitivity to gefitinib in resistant NSCLC cells. Several studies demonstrated that miRNAs are able to re-sensitize NSCLC cell lines to gefitinib through different molecular mechanisms. In particular, it was shown that miR-19a was downregulated in NSCLC cell lines with acquired resistance to gefitinib compared to sensitive cell lines and that its overexpression re-sensitized gefitinib-resistant NSCLC cells by targeting c-Met [28]. Also, miR-438-3p overexpression attenuated the acquired and intrinsic resistance to gefitinib in NSCLC cell lines through FAK, Akt, and Erk phosphorylation [29]. Moreover, miRNA-138-5p expression was reduced in PC9 gefitinib-resistant and H1975 cells, and its overexpression induced gefitinib response by targeting G protein-coupled receptor 124 [30]. Finally, upregulation of miR-133a-3p has been reported to reverse gefitinib resistance in NSCLC cells through the suppression of PI3K/AKT signaling [31]. Our results suggested that restoring miR-23c levels might be a potential therapeutic strategy in NSCLC carrying activating EGFR mutations with both intrinsic and acquired resistance to EGFR-TKIs. In this regard, miRNA-based therapeutic agents are in an early phase of clinical development and may potentially represent a strategy for overcoming cancer resistance mechanisms. However, several challenges limit clinical development, including the limited ability of miRNAs to penetrate target cells, the instability in the bloodstream, the off-target effects, and immune-related toxicity. Advanced delivery strategies, including lipid or polymeric nanoparticles, viral-based vectors, or exosomes, are under development to increase delivery to the tumor site and to reduce systemic toxicity [32]. However, a better understanding of the complex mechanisms underlying miRNA target regulation and the context-dependent role of miRNAs is pivotal for overcoming resistance development and optimizing precision medicine treatments.

Mechanistically, we demonstrated that IL-6R is a direct target of miR-23c and that the upregulation of miR-23c in gefitinib-resistant cells inhibited the IL-6R/STAT3 signaling pathway. We previously reported that miR-23c affected IL-6R/STAT3 signaling in breast cancer cell lines [12]. In lung cancer, the activation of the IL-6R/STAT3 pathway plays a significant role in tumor development and malignant progression by promoting cell proliferation, invasion, and EMT [33]. In agreement with this evidence, we observed that miR-23c mimic reduced EMT markers in resistant cells, suggesting a potential role of the IL-6R/STAT3 pathway in regulating migration and invasion of gefitinib-resistant cells.

Activation of alternative signaling pathways, including the IL-6R/STAT3 pathway, has been demonstrated to play a role in the acquired resistance to EGFR-TKIs. In this regard, EGFR-mutant NSCLC cells, but not EGFR wt cells, are able to activate a positive feedback loop that leads to STAT3 activation through IL-6R, thus significantly contributing to resistance to treatment [34]. In addition, secreted IL-6 in the tumor microenvironment may potentiate the intrinsic resistance to these drugs via paracrine activation of STAT3 [35]. For the first time, our study demonstrated that miR-23c mimic inhibited IL-6R expression and STAT3 activation in EGFR-mutant NSCLC cell lines resistant to gefitinib and re-sensitized cells to the drug.

More importantly, we demonstrated that the blockade of the IL-6R/STAT3 pathway with an anti-IL6R antibody significantly potentiated the effects of gefitinib in cell lines overexpressing miR-23c and resistant to the drug. It has been shown that treatment of gefitinib-resistant NSCLC cell lines with the EGFR-TKI afatinib can induce IL-6R signaling. The blockade of IL-6R protein combined with afatinib increased the sensitivity to the drug activation, thus bypassing EGFR inhibition [35]. In this regard, our results suggested that targeting miR-23c with the simultaneous blockade of IL-6R potentiated the growth inhibitory effects of gefitinib, counteracting the feedback loop leading to STAT3 activation.

Finally, we previously reported that EGFR activation downregulated miR-23c expression and secretion in the breast cancer microenvironment, suggesting a role of circulating miR-23c as a potential non-invasive biomarker [12]. Some studies have suggested the role of miRNAs as circulating biomarkers of response to targeted treatments and immunotherapy in lung cancer patients [36,37,38]. Here, we provided the rationale for further investigation of the role of circulating miR-23c in NSCLC patients carrying EGFR-activating mutations and candidates for EGFR-TKI treatment.

Our study lacks in vivo experiments. In this regard, few studies have analyzed the effects of miR-23c in vivo in cancer. One study demonstrated that miR-23c acted as a tumor suppressor in hepatocellular carcinoma both in vitro and in vivo [14], whereas another study showed that miR-23c did not inhibit tumor growth of prostate cancer cells in vivo, although a marked suppression of cell proliferation in vitro was observed [39], suggesting that different factors, including the genetic background of cancer cells and the tumor microenvironment, might affect miR-23c functions in vivo. Further studies are required to characterize the functional role of miR-23c in vivo in EGFR-mutant lung cancer. Nevertheless, our study provides information that might help to understand the relevant function of miR-23c in EGFR-mutant lung cancer cells resistant to gefitinib.

## 5. Conclusions

In conclusion, our data demonstrated that miR-23c plays a role in EGFR-mutant lung cancer cell lines with intrinsic and acquired resistance to EGFR-TKIs by regulating cell proliferation, migration, and invasion via the IL-6R/STAT3 signaling pathways. Importantly, we showed that the blockade of IL-6R potentiates the growth inhibitory effects of gefitinib in NSCLC cell lines resistant to the drug that overexpress miR-23c. Collectively, our study provides relevant information on the role of miR-23c in regulating resistance to EGFR-TKIs, paving the way for the development of new therapeutic approaches aimed at preventing or overcoming resistance in NSCLC patients carrying EGFR mutations.

## Data Availability

The data presented in this study are available in the article, in Supplementary Materials, and in Zenodo (10.5281/zenodo.19200908).

## Supplementary Materials

The following supporting information can be downloaded at: https://www.mdpi.com/article/10.3390/cells15121043/s1. Supplementary Figure S1: MiR-23c expression levels in NSCLC cells transfected with miR-23c mimic or inhibitor. Supplementary Figure S2: Effect of miR-23c inhibition on proliferation of the gefitinib-sensitive HCC827 NSCLC cell line. Supplementary Figure S3: Effect of miR-23c inhibition on gefitinib sensitivity in HCC827 cells. Supplementary Figure S4: Original immunoblotting images relative to epithelial–mesenchymal transition marker expression. Supplementary Figure S5: Original immunoblotting images relative to IL-6R expression and downstream signaling activation. Supplementary File S1. Candidate target genes of miR-23c (N = 342) listed according to descending AU score.

## Abbreviations

The following abbreviations are used in this manuscript:

ATCC	American Type Culture Collection
BSA	Bovine serum albumin
EGFR	Epidermal growth factor receptor
FBS	Fetal bovine serum
HGF	Hepatocyte growth factor
IL-6R	Interleukin 6 receptor
INC	Inhibitor negative control
NSCLC	Non-small-cell lung cancer
NTC	Non-targeting control
pSTAT3	Phospho-STAT3
STAT3	Signal transducer and activator of transcription 3
TKIs	Tyrosine kinase inhibitors
