# Supplementary material for: MiR-23c Regulates the Resistance to Gefitinib in EGFR Mutant Non-Small-Cell Lung Cancer Cells
Source: Cells. 2026 Jun 6;15(12):1043. doi: 10.3390/cells15121043 (PMC13296432; doi:10.3390/cells15121043)
Supplement: Supplementary file 1 [file cells-15-01043-s001.zip › cells-4259219-supplementary.pdf]

MiR-23c Regulates the Resistance to Gefitinib in EGFR mutant Non-Small-Cell Lung Cancer Cells

Supplementary material

Supplementary Figure S1. MiR-23c expression levels in NSCLC cells transfected with miR-23c mimic or inhibitor. The levels of expression of miR-23c in H1975 (a) and HCC827-GR (b) cells transfected with the miR-23c mimic or the non-targeting control (NTC) were measured by Real-time PCR. (c) MiR-23c expression levels in HCC827 cells transfected with miR-23c inhibitor or the inhibitor negative control (INC) measured by real-time PCR.
All data are presented as the mean ± SD from two experiments (* $p<0.05$ for comparison with control cells, two-tailed Student's t-test).

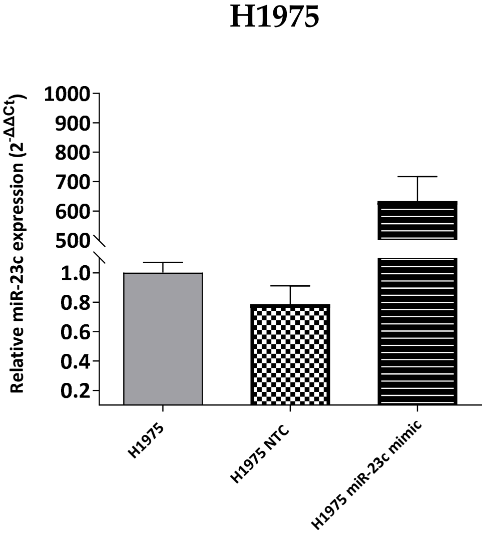

(a)

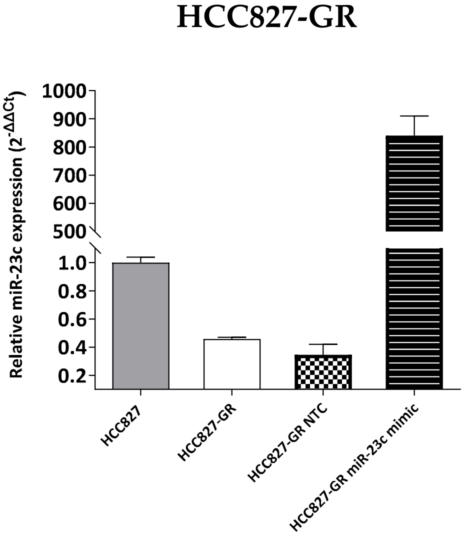

(b)

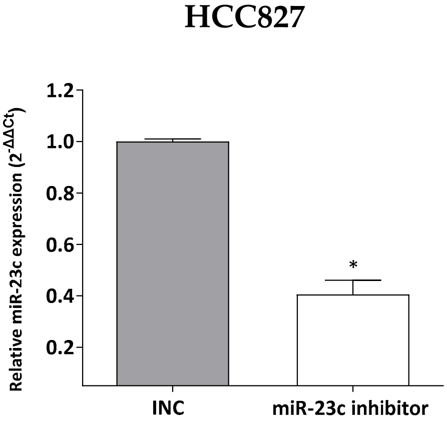

(c)

Supplementary Figure S2. Effect of miR-23c inhibition on proliferation of the gefitinib-sensitive HCC827 NSCLC cell line. Cell proliferation of HCC827 cells transfected with the miR-23c inhibitor compared to cells transfected with the inhibitor negative control (INC) was measured by MTT assay 72 h after transfection. Data are presented as the mean ± SD from at least two experiments (n=10) (*** $p<0.0001$ for comparison with control cells, two-tailed Student's t-test).

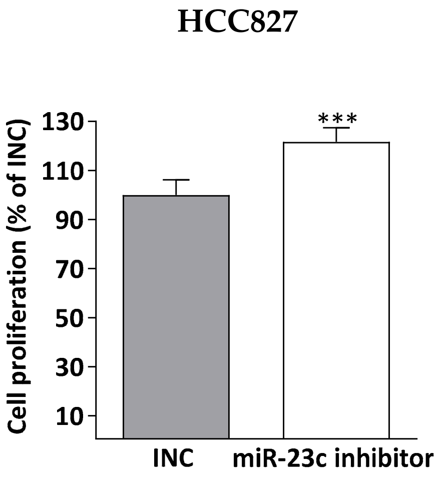

Supplementary Figure S3. Effect of miR-23c inhibition on gefitinib sensitivity in HCC827 cells. HCC827 cells were transfected with the miR-23c inhibitor or the INC and cell proliferation was measured by MTT assay after 72 h of treatment with gefitinib at the indicated doses. All data are presented as the mean ± SD from two independent experiments (* $p<0.05$ and *** $p<0.0001$ for comparison with cells transfected with the INC, two-tailed Student's t-test).

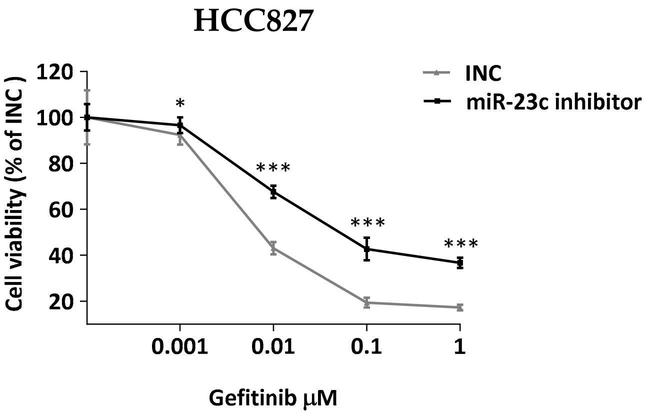

Supplementary Figure S4. Original immunoblotting images relative to epithelial mesenchymal transition markers expression. (a) Western Blot panels relative to figure 4c. (b) Western Blot panels relative to figure 4d. The original scans of the blots are shown. Squares indicate the images used for final figures.

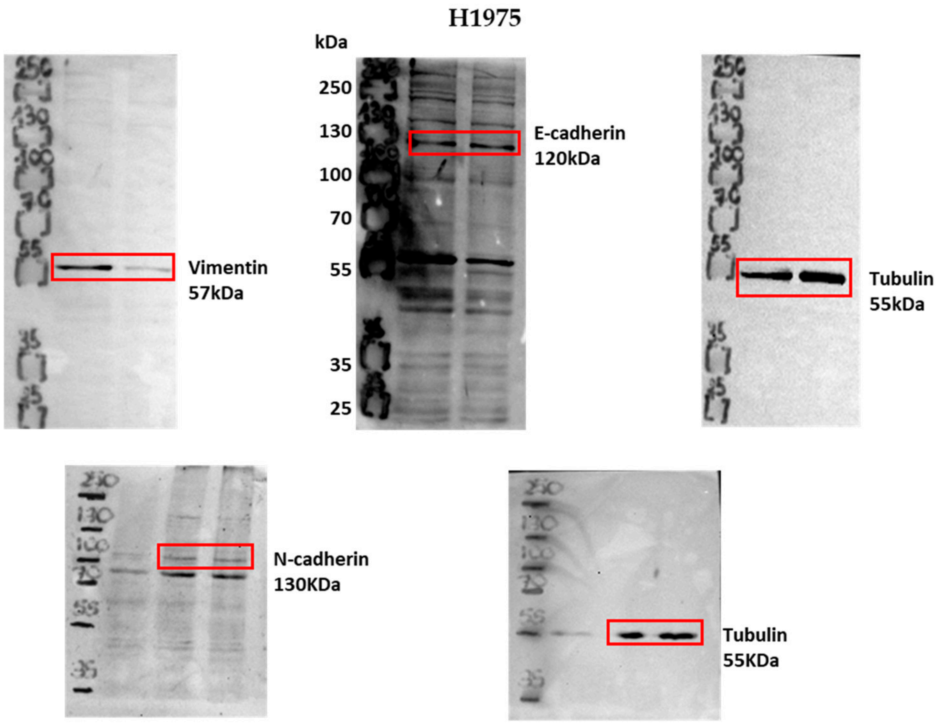

(a)

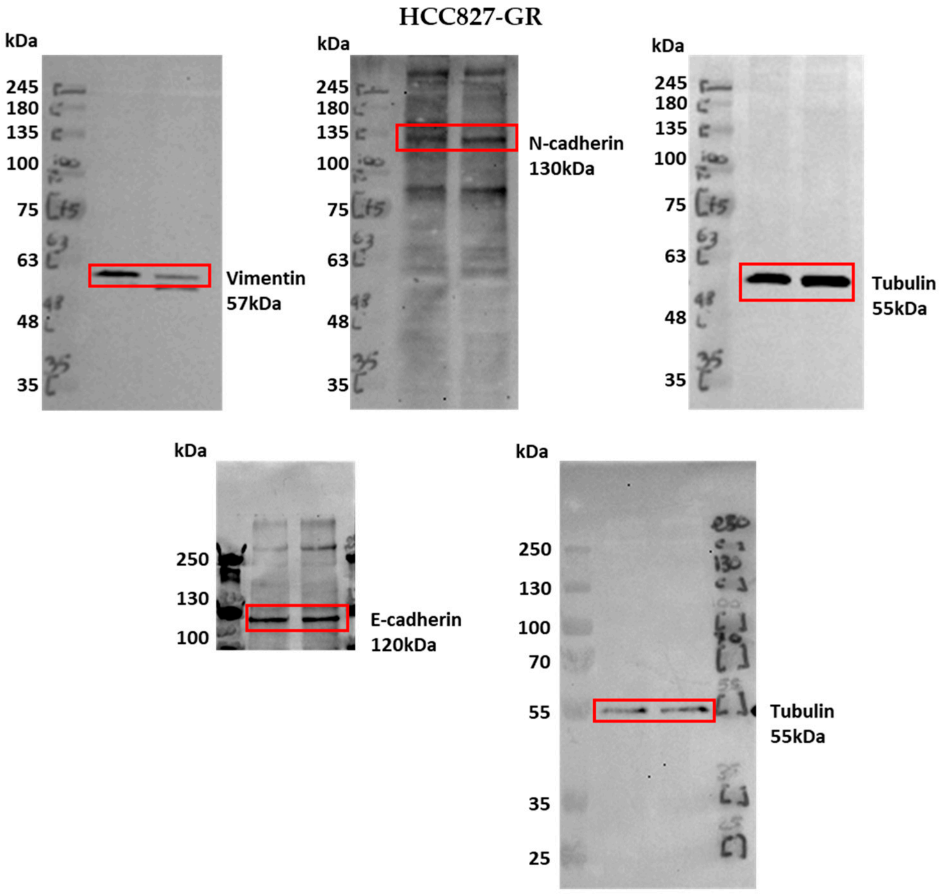

(b)

Supplementary Figure S5: Original immunoblotting images relative to IL-6R expression and downstream signaling activation. (a) Western Blot panels relative to figure 6c in the main text. (b) Western Blot panels relative to figure 6d in the main text. The original scans of the blots are shown. Squares indicate the images used for final figures. Densitometric analyses were reported in the figures 6c-d in the main text.

H1975

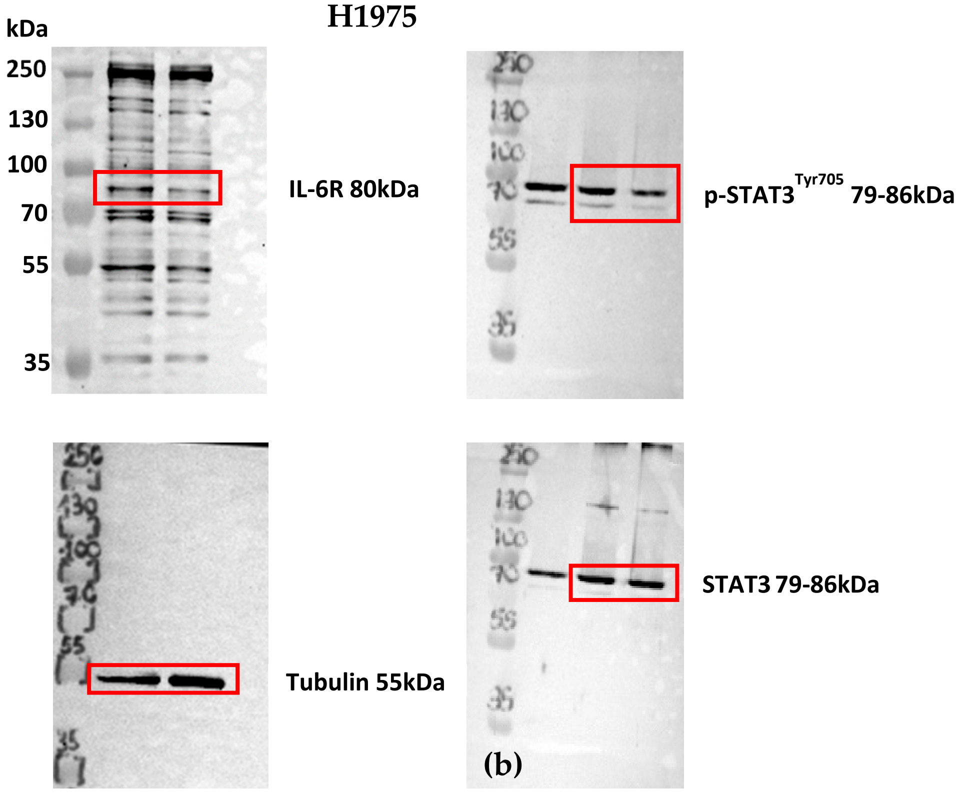

(a)

HCC827-GR

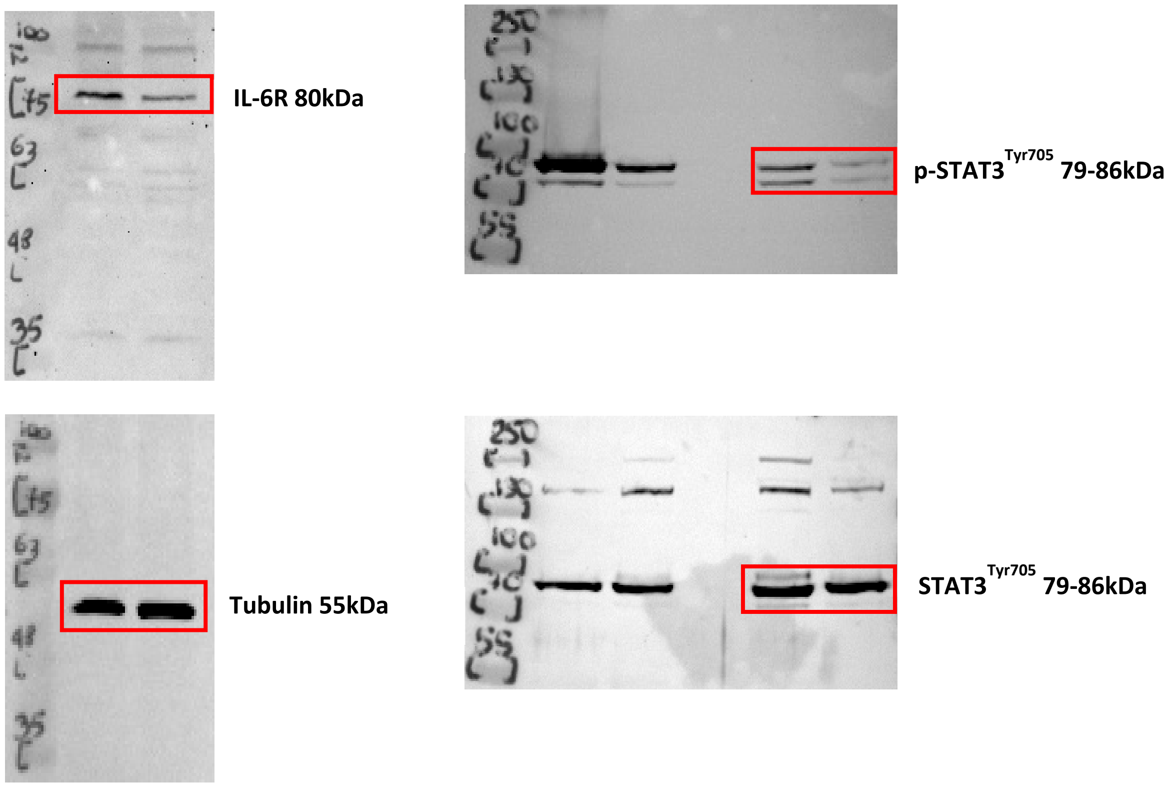

(b)
